# Atmospheric pollutants and their association with olive and grass aeroallergen concentrations in Córdoba (Spain)

**DOI:** 10.1007/s11356-020-10422-x

**Published:** 2020-08-13

**Authors:** Maria Pilar Plaza, Purificación Alcázar, José Oteros, Carmen Galán

**Affiliations:** 1 Chair and Institute of Environmental Medicine, UNIKA-T, University of Augsburg - Technical University of Munich (TUM) and Helmholtz Zentrum München, Neusässer Str. 47, 86156 Augsburg, Germany; 2 Department of Botany, Ecology and Plant Physiology, University of Córdoba (UCO), Córdoba, Spain; 3 Center of Allergy & Environment (ZAUM), Member of the German Center for Lung Research (DZL), Technische Universität München/Helmholtz Center, Munich, Germany

**Keywords:** Pollutants, Aeroallergens, Pollen, Olive, Grass, Climate change

## Abstract

Cumulative data indicate that pollen grains and air pollution reciprocally interact. Climate changes seem also to influence pollen allergenicity. Depending on the plant species and on the pollutant type and concentration, this interaction may modify the features and metabolism of the pollen grain. Previous results revealed a significant positive correlation between pollen and aeroallergen, even using two different samplers. However, some discrepancy days have been also detected with low pollen but high aeroallergen concentrations. The main aim of the present paper is to find how the environmental factors, and specially pollutants, could affect the amount of allergens from olive and grass airborne pollen. Pollen grains were collected by a Hirst-type volumetric spore trap. Aeroallergen was simultaneously sampled by a low-volume Cyclone Burkard sampler. Phl p 5 and Ole e 1 aeroallergen were quantified by double-sandwich ELISA test. The data related to air pollutants, pollen grains, and aeroallergens were analyzed with descriptive statistic. Spearman’s correlation test was used to identify potential correlations between these variables. There is a significant positive correlation between aeroallergens and airborne pollen concentrations, in both studied pollen types, so allergen concentrations could be explained with the pollen concentration. The days with unlinked events coincide between olive and grass allergens. Nevertheless, concerning to our results, pollutants do not affect the amount of allergens per pollen. Even if diverse pollutants show an unclear relationship with the allergen concentration, this association seems to be a casual effect of the leading role of some meteorological parameters.

**Electronic supplementary material:**

The online version of this article (10.1007/s11356-020-10422-x) contains supplementary material, which is available to authorized users.

## Introduction

Air pollution is a mixture of diverse particles and gasses in the air. Car emissions, chemicals from factories, dust, and also pollen grains and mold spores may be suspended as particles. Air pollutants, especially those related to climate change, will affect the immune reaction (Annesi-Maesano, 2016; Eguiluz-Gracia et al., 2020; Sheehan et al., 2017), when modifying the pollen grains, causing an inflammatory answer in the airways and an increased predisposition to aeroallergen exposure (Provost et al. 2014), such as asthma and allergic rhinitis (Noyes et al. 2009).

Atmospheric pollution plays a key role in all age set health (Anderson et al. 2013; Crouse et al. 2015; Fernández-Navarro et al. 2017). It has become obvious that urban individuals have more severe symptoms of respiratory allergies related with those living in rural areas (D’Amato et al. 2010). In cities, with high pollutant levels, biomass of vegetation and flowering seem to increase and therefore, rising airborne pollen concentration and thus pollinosis (Peden and Reed 2010; Schmidt 2016; Shea et al. 2008).

Numerous studies have investigated the effects of short-term exposure to air pollutants on respiratory symptoms and a positive association was found between exposure to ozone, carbon monoxide, nitrogen dioxide, sulfur dioxide, and particulate matter of 10 μm with asthma-related hospitalizations (Kim et al., 2015; Oduber et al., 2019; Oh, 2018; Zhang et al., 2015).

Effects of pollution on the worldwide bioaerosols are already known and studied (Bartra et al. 2007; Jenerowicz et al. 2012, Sedghy et al. 2018). This interaction can take place through a number of mechanisms; chemical pollutants could facilitate pollen allergen release, act as adjuvants to excite IgE-mediated responses, transform allergenic potential, and develop the expression of some allergens in pollen grains (Sedghy et al. 2018). On the other hand, atmospheric pollutants might cause the following direct effects on pollen (Sénéchal et al. 2015): diminution in viability and germination, variation of the physicochemical features of the pollen grain surface, modification in the allergenic potential, and adjuvant effect increasing their potential health risks. Pollutants and climate change affect not only pollen allergenicity (D’Amato et al., 2015; Naclerio et al., 2020), but also allergen concentration. Rogerieux et al. (2007) detected a decrease in allergen content in pollen samples treated with a mix of NO_2_/O_3_ or NO_2_/SO_2_. Short-term exposure of oak pollen to high concentrations of SO_2_ or NO_2_ significantly increases their fragility and disruption, leading to subsequent release of pollen cytoplasmic granules into the atmosphere (Ouyang et al. 2016).

Cumulative data indicate that pollen grains and air pollution reciprocally interact. Depending on the plant species and on the pollutant type and concentration, this interaction may modify the vitality, shape, size, physiologic features, and metabolism of the pollen grain (Malayeri et al. 2012). In recent studies, air pollution induced structural changes in macromolecules of mugwort pollen (Chen et al., 2020; Depciuch et al., 2016), which might contribute to an increment prevalence of allergic diseases in urban environs.

Global warming resulting climate change has been also causing indirect effects, such as an increase in the number of air pollutants as well as expansion of allergens (Cecchi et al. 2017; D’Amato et al. 2016; McMichael et al. 2006). Tashpulatov et al. (2004) found that increasing temperature determines higher allergen contents in birch pollen.

It has been shown that pollutants, gaseous, and/or particulate are also carried on allergenic particles as pollen or fungal spores (Behrendt et al. 1997). Moreover, air pollution possibly will have indirect effects by combining aeroallergens with some particles, such as diesel particles (Chehregani and Kouhkan 2008). In fact, some researchers use the neologism “polluen” (LAAIDI et al. 2002; Laaidi et al. 2011; Peltre 1998; Sénéchal et al. 2015) to point out this specific atmospheric material. It could contain water-soluble allergens or non-water-soluble ones and have many shapes and sizes. They can act as carriers of allergens and help their dispersion and access to the airways (D’Amato et al. 2007). Several researchers identified relevant allergens carried by airborne particles released from pollen of grass (Abou Chakra et al. 2012), ragweed (Pazmandi et al. 2012), and cypress (Shahali et al. 2009a), under rain or on in vitro treatment. Modification of pollen coating by air pollutants should be accounted for in further studies on the effect of pollution on germination and on allergenicity (Naas et al. 2016).

Additionally, air pollution and climate changes might make a greater expression of allergenic molecules in pollen grains attributable to adaptation of plants to abiotic stress (Mousavi et al. 2019; Shahali and Dadar 2018). Pollutants and climate changes are among the main plant stressors, especially in urban areas. Therefore, airborne pollen is today considered a sensitive indicator of plant climate change (Oteros et al. 2015; Recio et al. 2018). Numerous studies are focusing on airborne pollen concentration trends (Galán et al. 2016; Smith et al. 2009; Sofiev et al. 2015; Ziello et al. 2012) or in paleobotany studies that indicate how sensitive the plants are to temperature changes (Nolan et al. 2018).

These environmental factors could enhance the quantity and prompt chemical modifications of allergens, increase oxidative stress in the human organism, and trigger allergic reactions. Specially, air pollutants can play a role of adjuvants and modify the immunogenicity of allergenic proteins, whereas atmospheric abundance and human exposure to bioaerosol and aeroallergens are affected by climate change (Reinmuth-Selzle et al. 2017).

One of these stress inducers is ozone, known to induce NADPH increase in ragweed pollen, which in turn generates reactive oxygen species (Pasqualini et al. 2011).

Previous studies in Córdoba (Spain) showed year-to-year differences in timing and intensity of Poaceae and olive pollen season vs. aeroallergens (Plaza et al. 2016a, b). These results revealed a significant positive correlation between these two variables, even using two different samplers (Plaza et al. 2017). However, some aeroallergens in the absence of airborne pollen were recorded before and after the pollen season. Pollen allergen potency (PAP) (Galán et al. 2017) value is also pretty different between both pollen types; on average, PAP is 50 ρg/m^3^ in grasses but 15 ρg/m^3^ in olive. Some discrepancy days have been also detected with low pollen but high aeroallergen concentrations.

Córdoba is also among the 200 municipalities in Spain whose air contains higher levels of exposure to carcinogenic pollutants of industrial origin (http://www.juntadeandalucia.es/temas/medio-ambiente/emisiones/calidad.html).

The main aim of the present paper is to find how the environmental factors affect the amount of allergens in the airborne pollen of olive and grasses. We specially focus on the impact of pollutants, such as O_3_, NO_2_, and SO_2_ on the PAP of the pollen as they are suggested as key factors by several authors (Malayeri et al. 2012; Sénéchal et al. 2015; Shahali et al. 2009a). We also focus our attention to explain the term “unusual episodes,” i.e., the days during that the PAP is extremely high, because they could have a special clinical relevance.

## Methods

### Airborne pollen and aeroallergen sampling

In flowering season, Poaceae and *Olea* airborne pollen and their major aeroallergen proteins (Phl p 5 and Ole e 1) were sampled during three consecutive years (2012–2014) in Córdoba (37° 53′ 0″ N y 44° 5′0″ W; 123 m a.s.l.), a medium-sized city in the southwestern Iberian Peninsula.

Pollen grains were detected by a Hirst-type volumetric spore trap (Hirst 1952), with methodology suggested by the Spanish Aerobiology Network (REA) in Management and Quality Manual (Soldevilla et al. 2007) and the European Aerobiology Society (EAS) (Galán et al. 2014). At the same time, aeroallergen proteins were sampled by a low-volume Cyclone Burkard sampler (Emberlin 1995), following the method by Takahashi et al. (2001) and adapted by Moreno-Grau et al. (2006). Afterwards, aeroallergen particles were quantified by double-sandwich ELISA test (Plaza et al. 2016a, 2016b; Rodríguez-Rajo et al. 2011; Moreno-Grau et al. 2006; Arilla et al. 2002, 2005, 2006).

The pollen season was defined following suggestions from Velasco-Jiménez et al. (2013), considering the flowering intensity and flowering behavior. For olive pollen season, the first day started on which 50 or more pollen grains/m^3^ were recorded (Plaza et al., 2016b). For grass pollen season, the first day started on which 10 or more pollen grains/m^3^ were collected (Plaza et al., 2016a). The end date was, both cases, the last day with these daily values or less for five consecutive days.

We considered in the analysis some “unusual episodes,” days with particularly high pollen allergen potency (PAP—ρg/pollen grain), i.e., greater allergens per pollen than double the average.

### Meteorological data

Meteorological data were provided by the Andalusia Regional Government Agroclimatic Information Network, with station located on the outskirts of Cordoba City (coordinates 37° 51′ 25″ N, 04° 48′ 10″ W).

### Air pollution data

The Network for Monitoring and Control of Atmospheric Pollution in Andalusia, which is responsible for analyzing the presence of pollutants in the atmosphere, provided the pollution data. The Net was composed of 91 measuring stations and 12 meteorological towers and is responsible for detecting possible emergency situations.

Measurement item in relation to the atmospheric environmental standards include sulfur dioxide (SO_2_), carbon monoxide (CO), nitrogen dioxide (NO_2_), fine dust (PM_10_), and ozone (O_3_), which are measured once every hour. The daily average of these data from the nearest station to the samplers (Lepanto Station coordinates 37° 53′ 29″ N, 04° 45′ 51″ W) was used.

### Statistical analysis

The data related to air pollutants (SO_2_, CO, NO_2_, PM_10_, O_3_), pollen grains, and aeroallergens were analyzed with descriptive statistic. Spearman’s correlation test was used to identify potential correlations between these variables and meteorological parameters, considering the pollen seasons for each pollen type. Calculations were performed using the SPSS version 14 software package for Windows and R (R Core Team, 2013; RStudio Team, 2020).

## Results

**Fig. 1:**
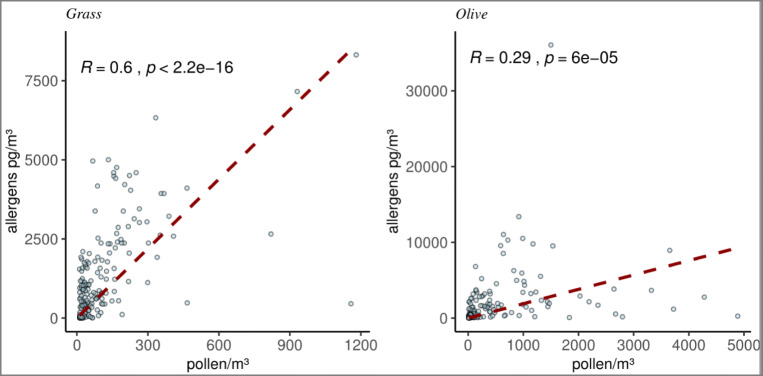
Pollen allergen potency in grass and olive

Aeroallergen and airborne pollen showed significant correlation (Fig. 1). However, some discrepancy days have been detected not only with low pollen and high aeroallergen concentrations but also with a PAP greater than double the average (mean in grasses: 44.58, mean in olive: 15.2). These “unusual episodes” largely matched in both olive and grasses in same dates (Table 1, figures in the Supplementary material).

**Table 1:** Unusual days during pollen season in studied period 2012–2014

Date	PAP grasses	PAP olive	Date	PAP grasses	PAP olive
2012-04-06	149.87	51.36	2012-04-23	119.16	–
2012-04-08	147.83	–	2012-04-24	213.63	–
2012-04-09	127.52	138.55	2012-04-25	93.44	106.24
2012-04-10	173.63	–	2012-04-29	1179.57	50.36
2012-04-11	378.69	–	2012-04-30	408.91	72.08
2012-04-13	325.99	35.99	2012-05-01	140.46	134.24
2012-04-15	278.83	31.05	2012-05-04	699.95	113.56
2012-04-16	235.40	161.88	2012-05-19	–	50.46
2012-04-18	129.87		2013-04-25	138.29	–
2012-04-19	375.44		2013-04-30	89.75	–
2012-04-20	91.93	63.28	2014-04-11	119.84	707.95
2012-04-21	191.29	–	2014-06-08	–	87.49
2012-04-22	142.68	–	2014-06-11	–	85.11

**Fig. 2:**
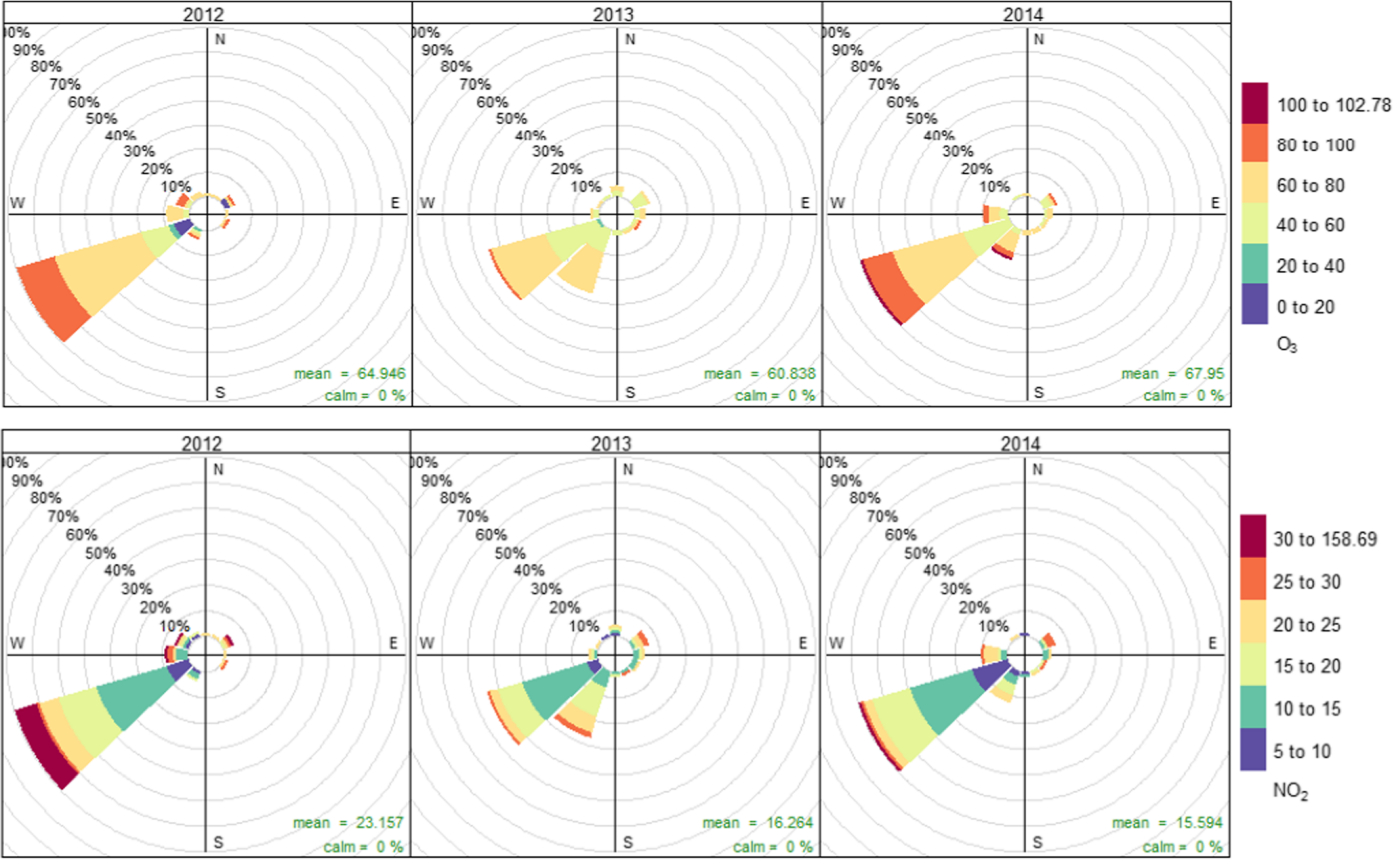
Frequency of days with the highest percentage of pollutants O_3_ and NO_2_, for each year and the prevailing wind direction

The average levels of SO_2_, PM_10_, NO_2_, CO, and O_3_, during 2012–2014, are shown in Table 2. Except for NO_2_, the average measures from all pollutants are lower during 2013 and higher percentages on days with great values were collected for all the pollutants during 2012 (Fig. 2). The highest concentrations in all cases are with a southwest wind. Regarding the daily pollutant concentration, it seems to be higher during the spring season (from 04-01 to 05-15) for CO and NO_2_ than during the early summer (from 05-16 to 07-01), with greater concentration for SO_2_, PM_10_, and O_3_ (Supplementary material).

**Table 2:** Average values of pollutants during the pollen season in period 2012–2014. SD = standard deviation. SE = standard error of the mean

	Statistics	SO_2_ (μg/m^3^)	PM_10_ (μg/m^3^)	NO_2_ (μg/m^3^)	CO (μg/m^3^)	O_3_ (μg/m^3^)
2012	Mean	5*.*33	21*.*36	23*.*16	466*.*52	64*.*95
	Maximum	14.54	128.32	158.69	913.78	97.31
	Minimum	3.14	1.00	7.99	87.01	6.69
	SD	1.44	16.68	26.87	287.69	21.20
	SE	0.15	1.73	2.80	29.99	2.21
2013	Mean	3*.*28	18*.*81	16*.*26	252.99	61*.*12
	Maximum	5.66	37.28	29.11	521.24	87.48
	Minimum	2.20	2.98	6.85	40.64	36.99
	SD	0.60	7.74	5.00	128.75	9.54
	SE	0.06	0.81	0.52	13.42	0.99
2014	Mean	4*.*31	20*.*37	15*.*59	427*.*44	67*.*95
	Maximum	5.27	36.35	31.39	661.0	102.78
	Minimum	3.40	5.95	5.76	184.2	44.99
	SD	0.45	7.46	6.12	110.97	12.85
	SE	0.05	0.77	0.64	11.57	1.34

Tropospheric ozone (O_3_) shows high levels in our study area, as 40 days during the pollen season period from 2012 to 2014 exceeded the average value limit 160 μg/m^3^ in an hour, concentration with registered negative health effects (WHO, 2000). These days are concentrated especially in June between 16.00 and 17.00. Regarding nitrogen dioxide (NO_2_), the limit value per hour has been exceeded only in the 18th of June 2012 (from 9.00 to 10.00) with measures greater than the reference limit value, 200 μg/m^3^.

**Fig. 3:**
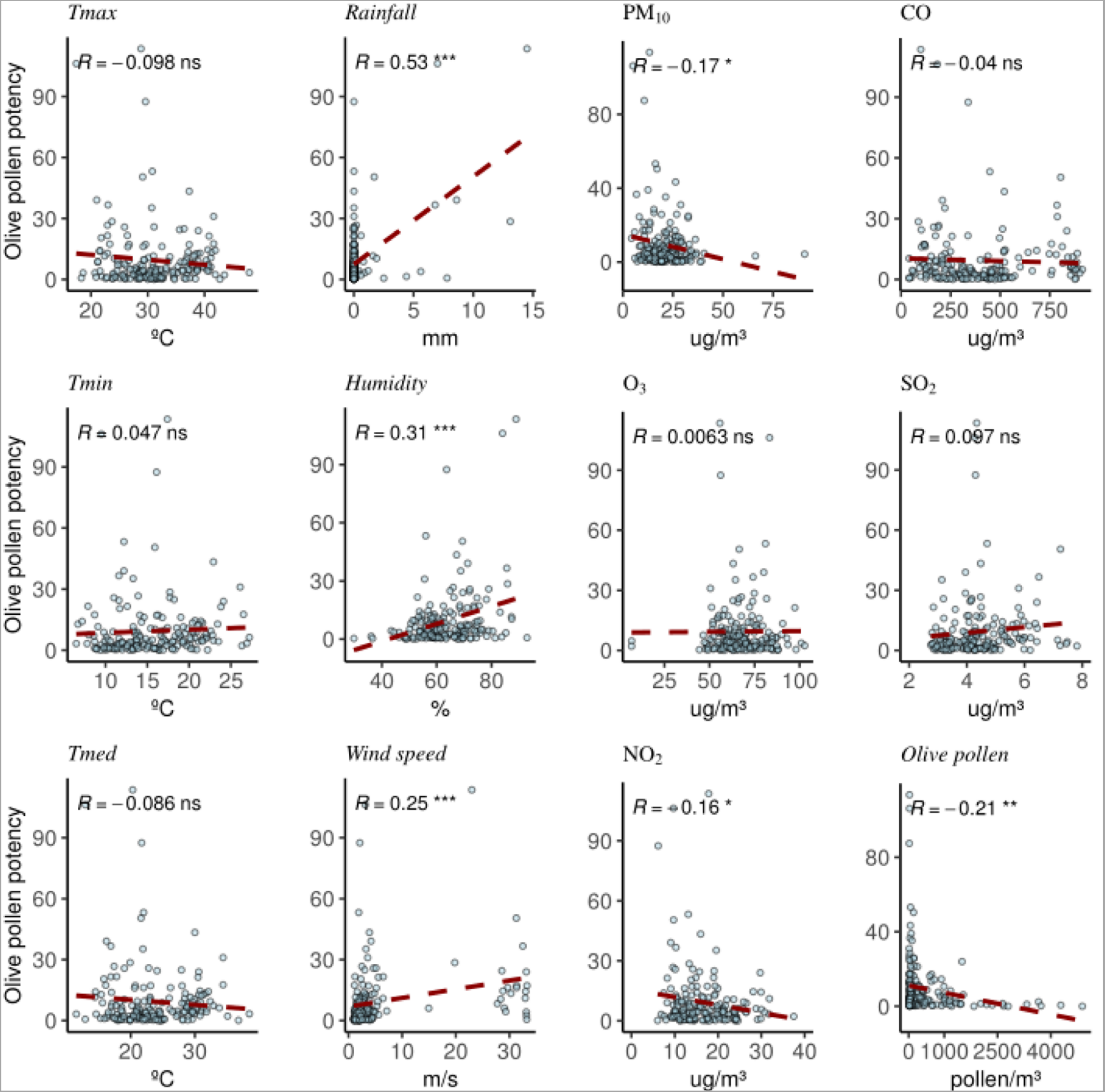
Olive PAP correlation with the different pollutants and meteorological parameters

**Fig. 4:**
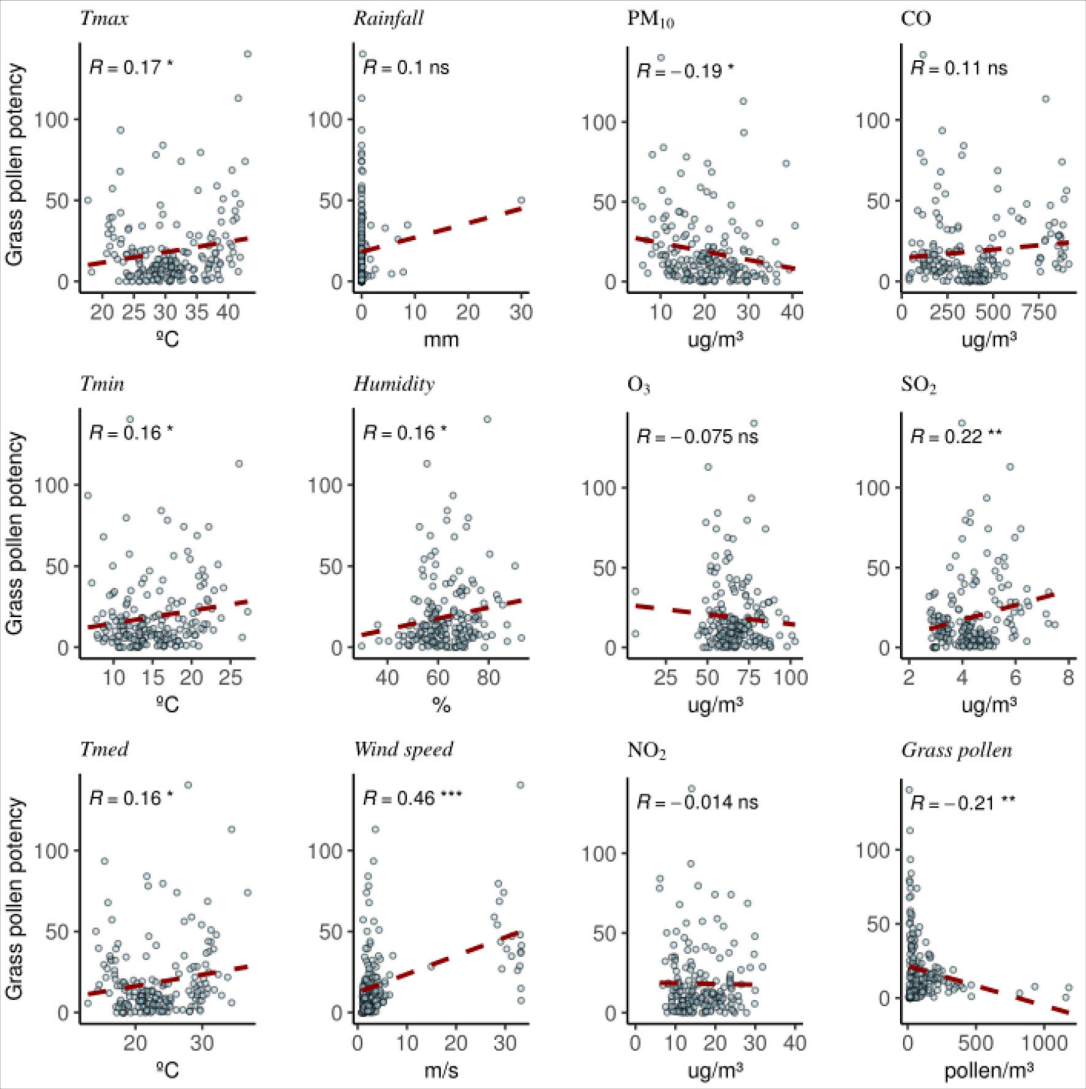
Grass PAP correlation with the different pollutants and meteorological parameters

Concerning the correlation between biological particles and pollutants, it has been observed that PAP and all the considered parameters have a vague relationship in both pollen types (Figs. 3 and 4). None of the pollutant shows a significant correlation for both pollen types, but PM_10_ seems to be lower when high PAP is reached. Allergen concentration is higher in relation to pollen when the relative humidity and rainfall increase, and so, it coincides with days with low pollen concentration and PM_10_.

Since we observe large differences in sporadic days, we present two groups, one that includes the days with expected values and the other with non-normal values. And we investigated the correlation of these two groups with the different variables.

**Fig. 5:**
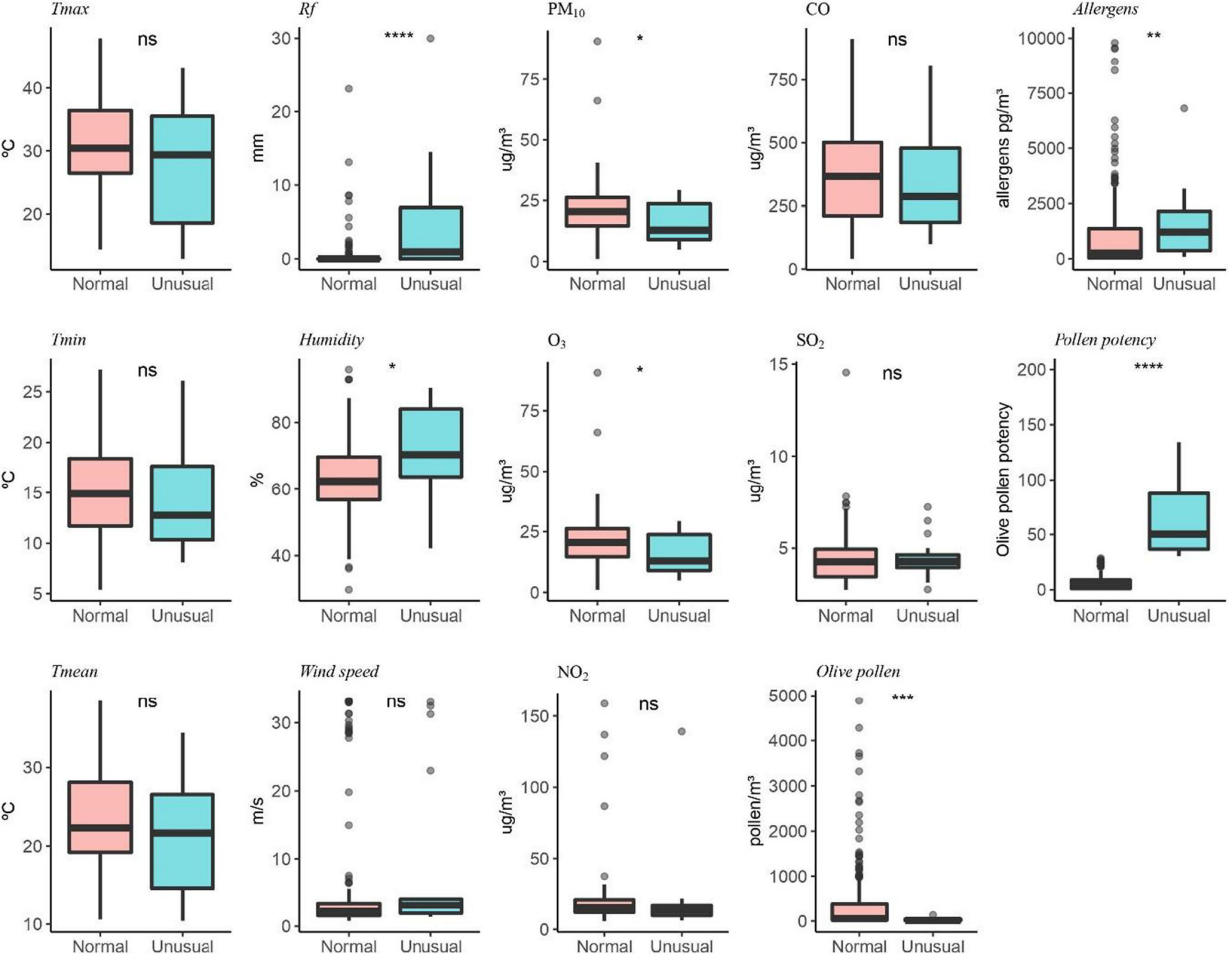
Comparison between main weather parameters and pollutants in normal and unusual episodes recorded during olive pollen season. *T* test was used ****p < 0.05; ***p < 0.001; **p < 0.0001; *p < 0.00001

**Fig. 6:**
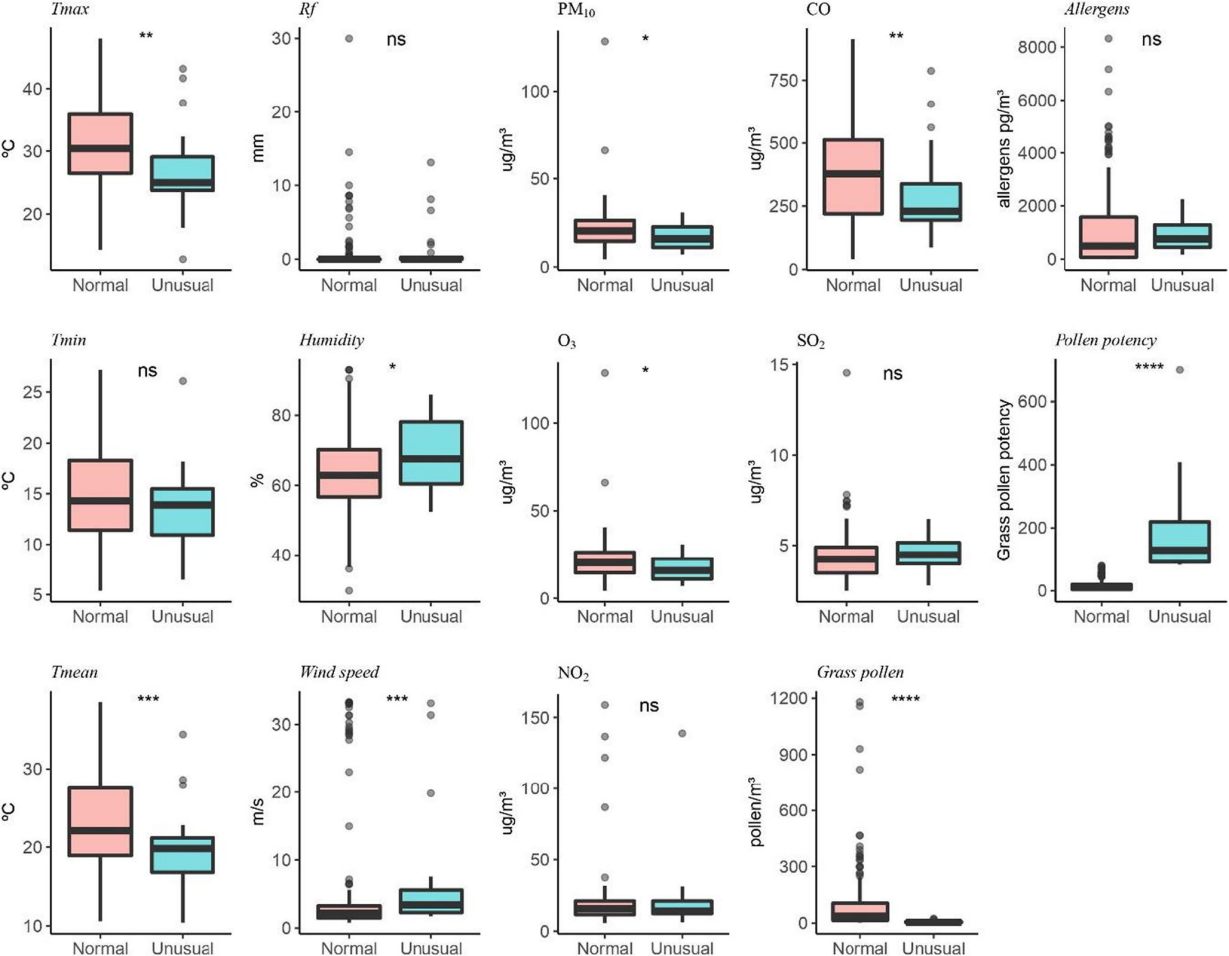
Comparison of main weather parameters and pollutants in normal and unusual episodes recorded during grass pollen season. *T* test was used ****p < 0.05; ***p < 0.001; **p < 0.0001; *p < 0.00001

Figures 5 and 6 show the different parameters studied separately in normal days with respect to the so-called unusual episodes (high PAP) in order to see possible differences between them. These events seem to occur in rainy or high humidity days, since they coincide with rainy days 70% of the times. The results indicate that in both olives and grasses, the parameters that have a significant relationship to the ratio allergens/pollen are O_3_ and PM_10_, though this correlation is negative since it has just been shown that PM_10_ is low when aeroallergens are high. Regarding meteorological parameters, humidity has significant correlation in both pollen types and temperature just with grasses. We also observed that humidity is especially relevant in unusual days that can be explained as rainy highly humid days with low pollen concentrations due to atmospheric washing but with greater allergen dispersion. In the case of grasses, a significant connection with temperature is found, probably due to the difference in PAP by grass species along the season. The connections of O_3_ and PM_10_ with PAP can be the result of the impact of weather conditions.

## Discussion

Pollution seems to produce several noxious effects on pollen grains (Oduber et al. 2019; Sénéchal et al. 2015). In most species, its pollen in urban areas is more allergenic than pollen in areas less exposed to pollution (D’Amato et al., 2010; Guarnieri and Balmes, 2014), even though some controversial results have been stated, where air pollution exposure does not seem to increase the general risk of sensitization to allergens (Berger et al., 2020; Gruzieva et al., 2012). Nevertheless, all of these studies emphasize the importance of integration environmental factors in the development of allergic diseases (Sbihi et al., 2019).

Most of the studies use correlation analysis between both particles; however, this analysis is not the most accurate for this study since we worked with sporadic days. For that reason, we define unusual days, with unexpected allergen concentration per pollen grain.

Regarding our study, there is a significant positive correlation between aeroallergens and airborne pollen concentrations, even using different samplers (Plaza et al. 2017) in both pollen type detections, so allergen concentrations could be explained with the pollen concentration. On the other hand, the days with unlinked events (unusual episodes) coincide between olive and grass allergens. Nevertheless, the role of pollutants is not shown in a clear way but does not seem to have any influence during the unusual episodes, since only O_3_ and PM_10_ have a correlation but this is negative. Both O_3_ and PM_10_ could be the reflection of rainy days with low amount of particles in the air (Yoo et al., 2014), as O_3_ is especially low during cloudy days.

Even if we do not find clear facts of the role of pollutants in free allergens, it is known that elemental composition of pollen is very often modified by pollution. Chemical changes on pollen grains are detectable when comparing pollen (*Thuja orientalis*) from polluted and unpolluted sites (Rezanejad 2009). In this study, it is suggested that some plant defense mechanisms are initiated in pollen affected by airborne particulate matter, adjusting their metabolism so that minimum damage is done due to air pollutants. Such as, the pollen grains collected from polluted zones are smaller and more fragile compared with the low polluted ones. The exine disruption seems to be faster and higher in polluted pollen grains (Duhoux 1982; Shahali et al. 2009b), which can facilitate the release of internal particles such as allergens (Behrendt et al. 1997; Sénéchal et al. 2015). This could mean that in urban areas, having more fragile pollen grains, more allergens are released (Lacroix 2005; Motta et al. 2006) and contribute to an increased pollen allergenicity (Costa et al., 2019) when are exposed to traffic-related air pollutants such as NO_2_ and O_3_. For example, *Platanus orientalis* pollen became swollen after several hours of fumigation with NO_2_, or SO_2_ (Lu et al. 2014). However, others found no differences between polluted and non-polluted pollen (Kanter et al. 2013). That discrepancy may be caused from differences in the studied pollen grains and the variable amounts of gas pollutants used. Moreover, it has been shown that the humidity also affects pollen in this regard (Buters et al. 2015; Plaza et al. 2016b). However, according to our results, unusual days with low pollen concentration but high allergen concentration do not take place during high peaks of pollutants in the atmosphere.

On the other hand, different modifications induced by air pollution have been studied at protein level. Pollutants play a role in the difference of protein amount and/or the presence or absence of proteins in comparative extracts. For instance, Shahali et al. (2009b) found a reduction in total protein amount on polluted sites as well as a clear decrease of the allergens. On contrast, after exposing different pollen grains to SO_2_, NO_2_, and O_3_, it shows the most important release of protein material (Thomas et al. 1996) with the most concentrated rate of dust. In other investigations, a significant decrease was found in the protein content in a pollen type exposed to NO_2_ independently and those exposed to SO_2_ and NO_2_ together (Bist et al. 2004). According to Ribeiro et al. (2017), this heterogeneity seems related, at least for some changes, to differences in pollutants (NO_2_ and O_3_) and interspecies variations. In another investigation, the O_3_ effects on *Pinus* pollen allergenicity have been reported (García-Gallardo et al. 2013), where the highest levels of specific IgE were found with highest O_3_ levels (45.90 μg/m^3^) but with lower values for other pollutants. It may be determined that pollen allergenicity and viability in some pollen type increased when vegetation is under elevated O_3_ conditions (Pasqualini et al. 2011). In our study, both O_3_ and PM_10_ seem to have some negative influence on the allergen concentration detected during unusual episodes. One possible cause of this correlation could be that the allergens transported by pollutants (polluen) are not well detected by the immunological test carried out, because they change in some way their protein structure and/or post-translational modifications affecting allergen recognition. In this regards, Rogerieux et al. (2007) detected a decrease in allergen concentration exposing pollen under gaseous pollutants (O_3_ and NO_2_) during only 4 h. Moreover, both O_3_ and PM_10_ could be the reflection of rainy days with low amount of particles in the air, as O_3_ is especially low during cloudy days. Regarding NO_2_, a study showed that its influence depends on the type of analyzed pollen (Chassard et al. 2015), perhaps because the exposure of pollen grains to other pollutants changes its structure (Ribeiro et al. 2017).

According to these statements, it is important to study the effect of all the pollutants at the same time, not independently, since the increase of one pollutant affects the plants in more or less virulence according to the amount of other contaminant particles in the atmosphere.

Several studies in urban-polluted air (Ghiani et al. 2012; Wang et al. 2010) showed that some airborne antigens were mainly adsorbed to combustion particles which can thus fly as depots on diesel exhaust particles (DEP) generated from transportation (Namork et al. 2006). They can also be associated with the presence of rainwater in polluted zones (Wang et al. 2013). Our results showed also an important and significant correlation between aeroallergens and humidity. The effect of humidity on pollen is supposed to facilitate the release of cytoplasmic granules and allergens (Buters et al. 2015; D’Amato et al. 2010). However, the humidity causes the deposition of pollen grains as they increase in weight (Barnes et al. 2001; Bartková-Ščevková 2003), explaining the rise in allergens per pollen detected. Moreover, pollen grains under extreme conditions could produce more allergens as a response to make sure pollination (Moreno-Grau et al., 2016; Plaza et al., 2016a). In the particular case of grasses, there is a sequence in the PAP depending on the species, and this can be observed in significant differences in seasonal variables such as temperature (Bruffaerts et al., 2018; García-Mozo, 2017; Ščevková et al., 2020).

Additionally, like temperature variations just as barometric ones are important in pollen release and air particle interactions, water is also a main point, specifically in inner pollen subparticle dispersion. According to Schäppi et al. (1999), light rain increases the dispersion by 20%, since it modifies shape and size of pollen. Depending on pollen season and meteorological conditions, the water content in pollen grains will be intensely influenced. On the other hand, the relative effect of the rainfall washout on the air pollutant concentrations results in a decrease of PM_10_, SO_2_, and CO in the atmosphere, while NO_2_ and O_3_ may increase due to opposing influences of lightning-generation or vertical mixing from the troposphere (Yoo et al., 2014).

Even with the positive tendency in the incidence of allergic sensitization in urban places, the factors that justify this phenomenon have not been settled so far. Though, accumulating evidence shows that climate change and pollutants seem to play a part as vegetal stressors, altering the allergenic potential of pollen particles. Their implication in allergy illnesses is previously described in some studies for different regions and pollen types (Bacsi et al. 2006; Kim et al., 2015, 2016). It has been shown that their presence in the air increases the aeroallergen bioavailability. It is important to consider that pollutants might be pollen allergy-initiating and/or facilitating by themselves. Although associations between air pollution and airborne pollen charge are still debated (Annesi-Maesano et al. 2012), experimental works seem to demonstrate that combined effects of atmospheric pollutants and pollen are very unfavorable to allergic people.

Air pollution coincides with the existing increase in the prevalence of allergic diseases (Motta et al. 2006). Our results indicate that the pollutants may not affect the concentration of allergens in the air in a city like Cordoba, where pollution values are not very high (Junta Andalucia, 2014). Nevertheless, it seems that there is consensus between different studies where there is a positive correlation between the increase of pollutants and the increase of symptoms in the population (Bacsi et al. 2006; Burte et al. 2018; Cabrera et al. 2018; Kim et al. 2016).

To better understand the complex relations between vegetation, atmosphere, mechanisms of pollen rupture, and weather conditions just as the effect on health, we need more standardized studies with different pollen types and diverse pollutant doses. Since, pollen response is not equal in front of the pollution and there are evidences that its susceptibility to pollutants varies according to the plant species.

Moreover, the effects of air pollution on the dispersion and allergenicity of these subparticles are awhile not clearly explained, because of the extremely complex pollen-pollutant interactions.

Therefore, it would be convenient to take into consideration this stress-induced increase in pollen particles by pollutants as a reliable environmental factor in respiratory allergy epidemiology.

## Conclusion

Outdoor aeroallergen is mostly due to pollen, but the relative humidity in the air could increase amount of allergens per pollen. Unusual episodes occur in Córdoba during rainy days with high relative humidity, decreasing pollen concentration, O_3_ and PM_10_.

Attending to our results, pollutants do not affect the amount of allergens per pollen in the studied city. Even if different pollutants show a vague relationship with the allergenic content of pollen, this relationship seems to be a casual effect of the leading role of some meteorological parameters. Further researches should shed light on the molecular interaction between pollutants and pollen grains, integrated with possible negative influenced in real-life allergic symptoms.

## Electronic supplementary material

ESM 1 (DOCX 308 kb)
