# Correction to: Atmospheric pollutants and their association with olive and grass aeroallergen concentrations in Córdoba (Spain)

**DOI:** 10.1007/s11356-021-14749-x

**Published:** 2021-06-14

**Authors:** Maria Pilar Plaza, Purificación Alcázar, José Oteros, Carmen Galán

**Affiliations:** 1 Chair and Institute of Environmental Medicine, UNIKA-T, University of Augsburg - Technical University of Munich (TUM) and Helmholtz Zentrum München, Neusässer Str. 47, 86156 Augsburg, Germany; 2 Department of Botany, Ecology and Plant Physiology, University of Córdoba (UCO), Córdoba, Spain; 3 Center of Allergy & Environment (ZAUM), Member of the German Center for Lung Research (DZL), Technische Universität München/Helmholtz Center, Munich, Germany

**Correction to: Environmental Science and Pollution Research (2020) 27:45447–45459**

10.1007/s11356-020-10422-x

The article Atmospheric pollutants and their association with olive and grass aeroallergen concentrations in Córdoba (Spain), written by Maria Pilar Plaza, Purificación Alcázar, José Oteros and Carmen Galán, was originally published electronically on the publisher’s internet portal on 13 August 2020 without open access. With the author(s)’ decision to opt for Open Choice the copyright of the article changed on 25 May 2021 to © The Author(s) 2021 and the article is forthwith distributed under a Creative Commons Attribution 4.0 International License, which permits use, sharing, adaptation, distribution and reproduction in any medium or format, as long as you give appropriate credit to the original author(s) and the source, provide a link to the Creative Commons license, and indicate if changes were made. The images or other third party material in this article are included in the article’s Creative Commons license, unless indicated otherwise in a credit line to the material. If material is not included in the article’s Creative Commons license and your intended use is not permitted by statutory regulation or exceeds the permitted use, you will need to obtain permission directly from the copyright holder. To view a copy of this license, visit http://creativecommons.org/licenses/by/4.0.

The Original article has been corrected.

