# Supplementary material for: Atmospheric pollutants and their association with olive and grass aeroallergen concentrations in Córdoba (Spain)
Source: Environ Sci Pollut Res Int. 2020 Aug 13;27(36):45447–59. doi: 10.1007/s11356-020-10422-x (PMC8197725; doi:10.1007/s11356-020-10422-x)
Supplement: Supplementary file 1 — (DOCX 308 kb) [file 11356_2020_10422_MOESM1_ESM.docx]

**SUPLEMENTARY MATERIAL**

*
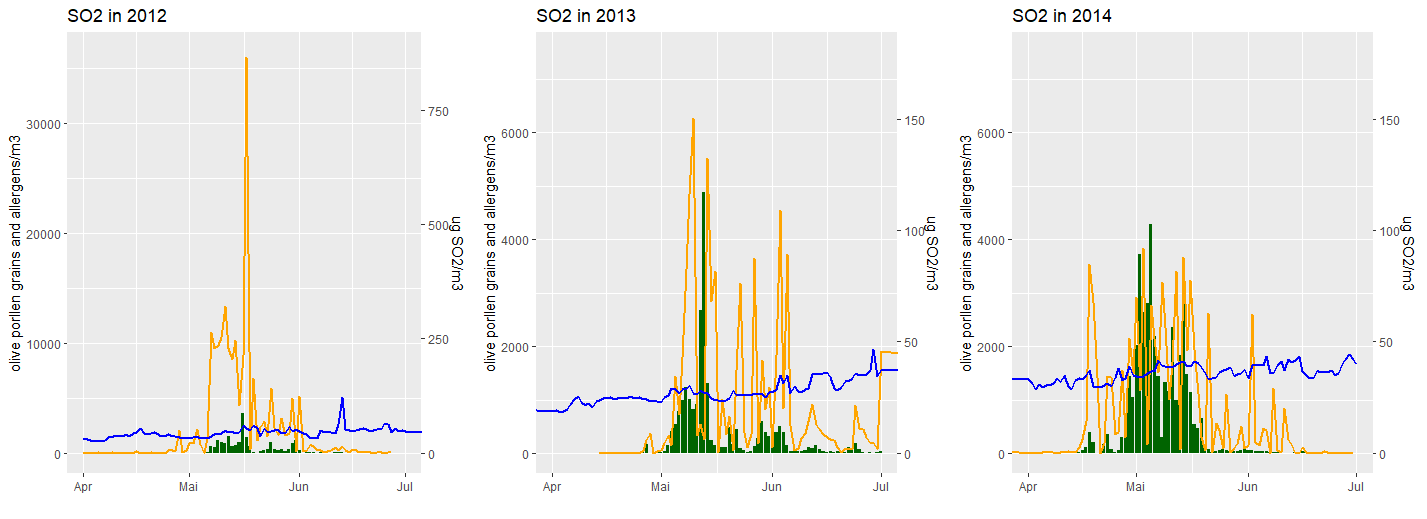

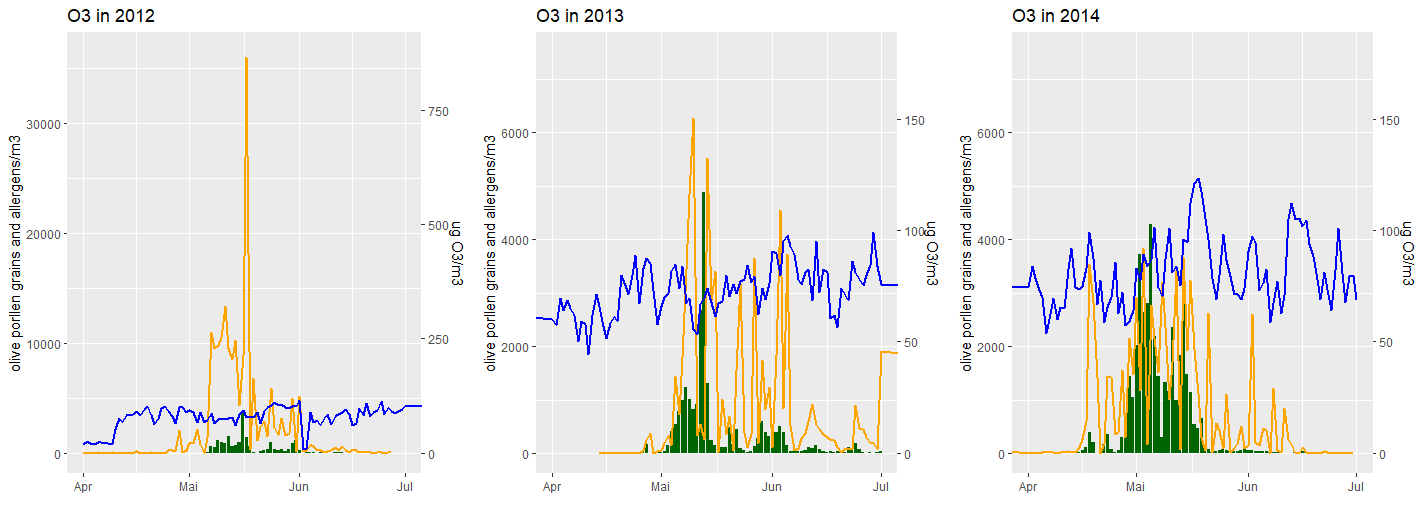

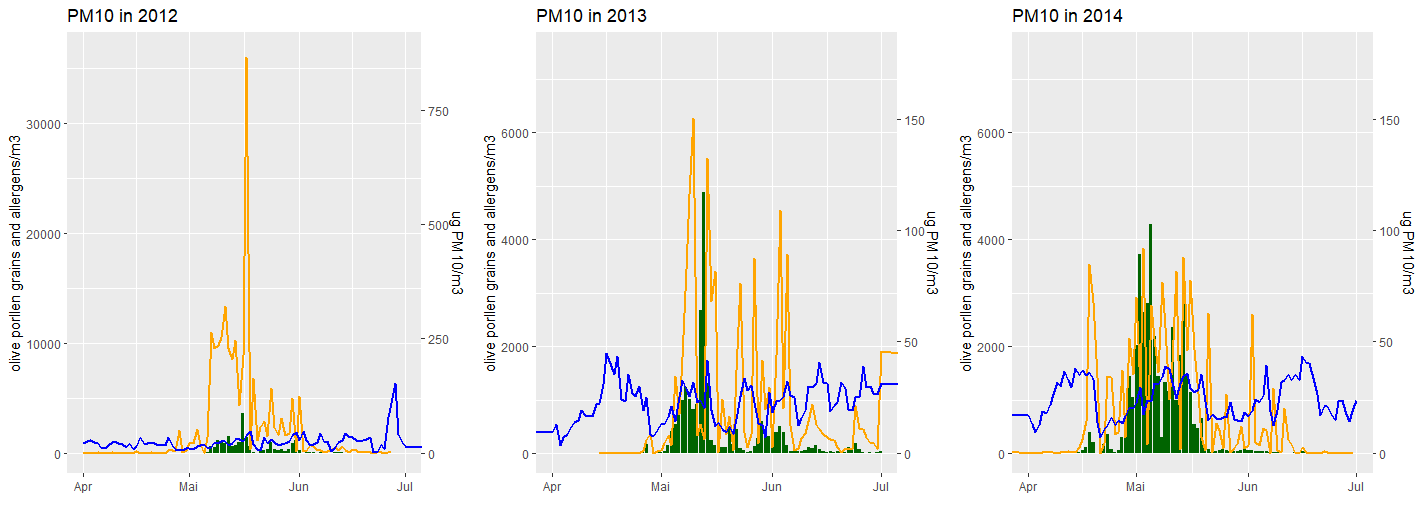

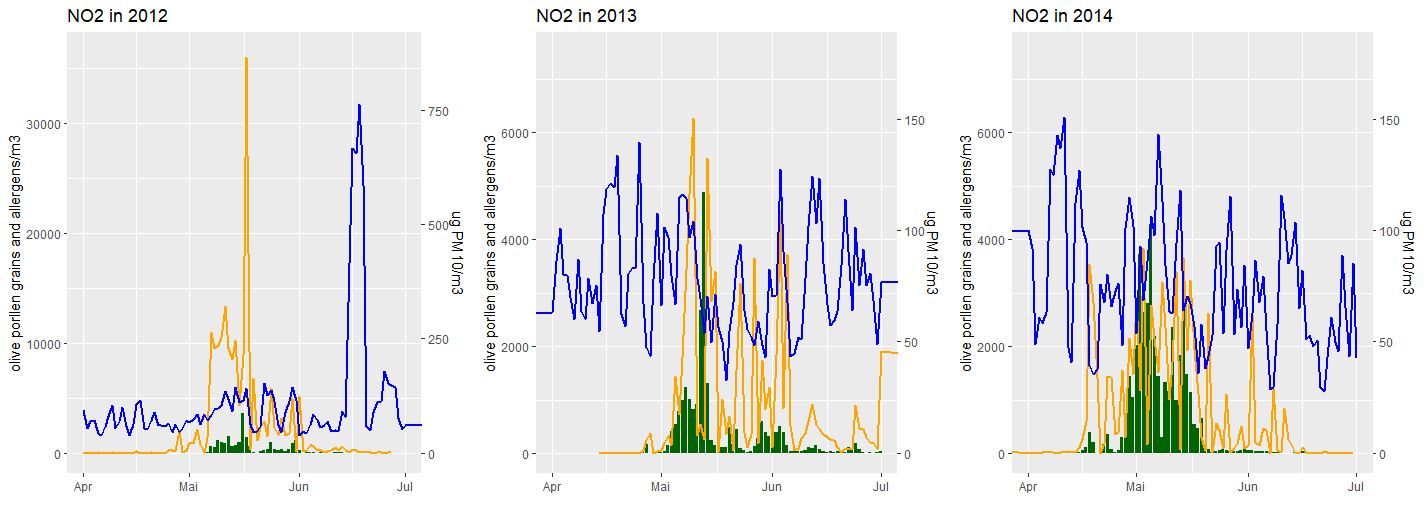

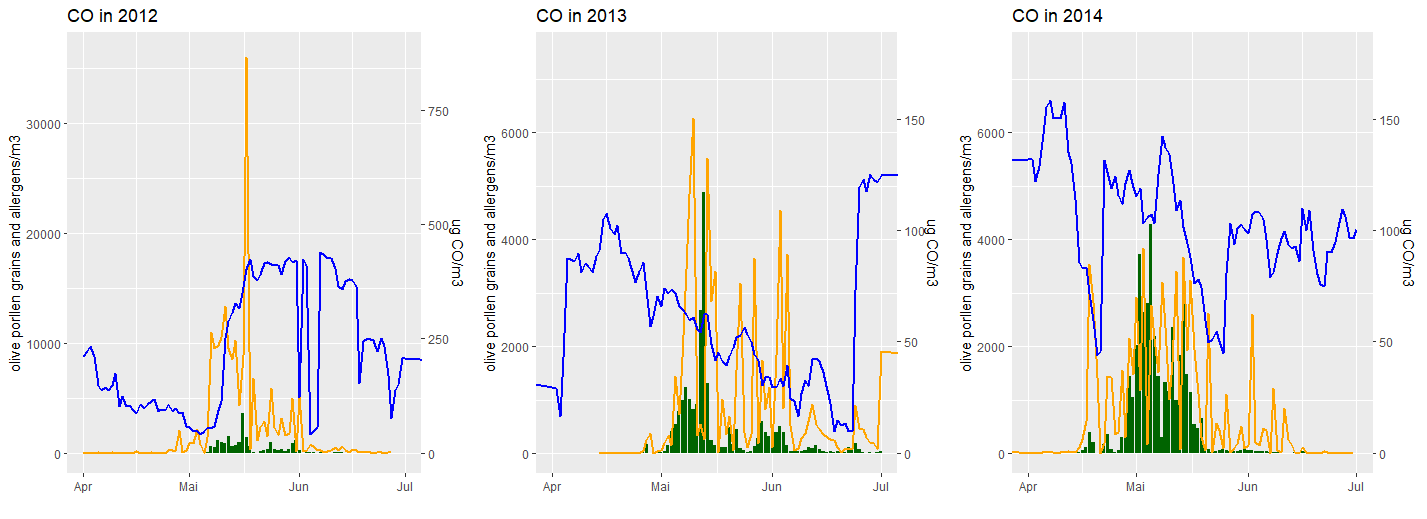
*

*Figure A. Olea Pollen (grains/m^3^) in green, Ole e 1 concentration in orange and SO_2,_ O_3,_ PM_10_, NO_2_ and CO pollutants concentration (in blue) during the studied period in Córdoba*

*
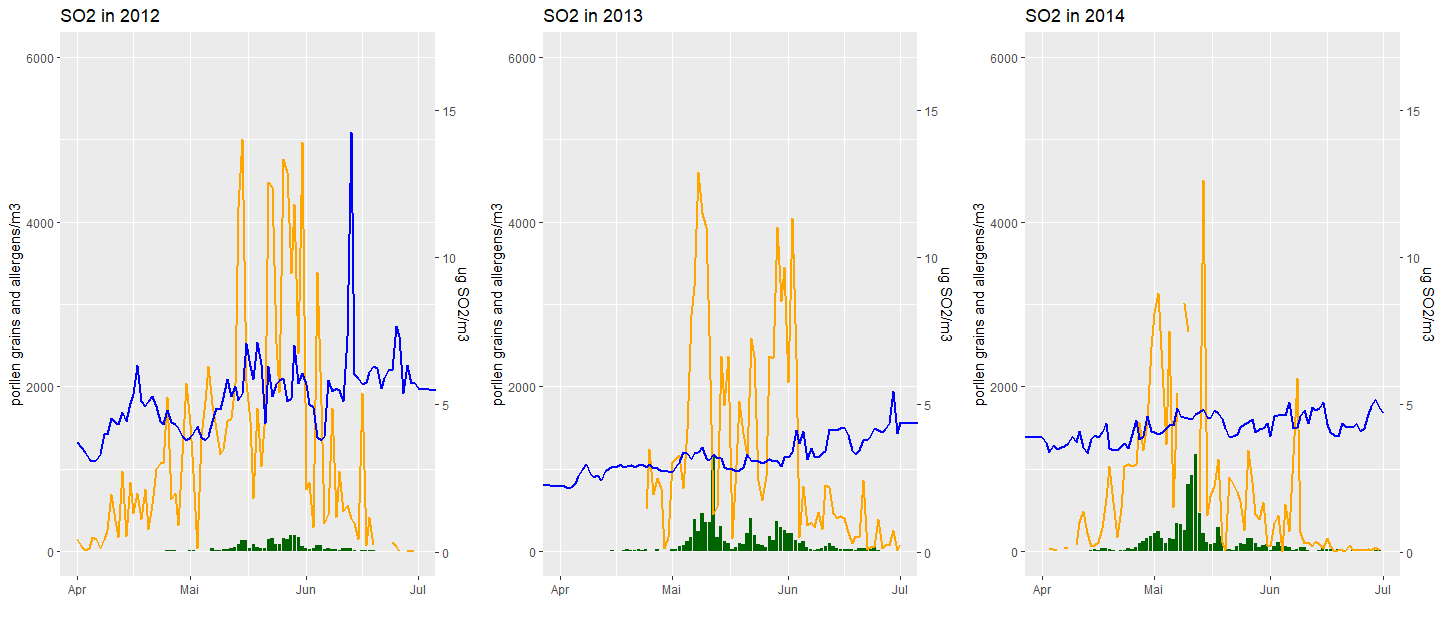
*

*
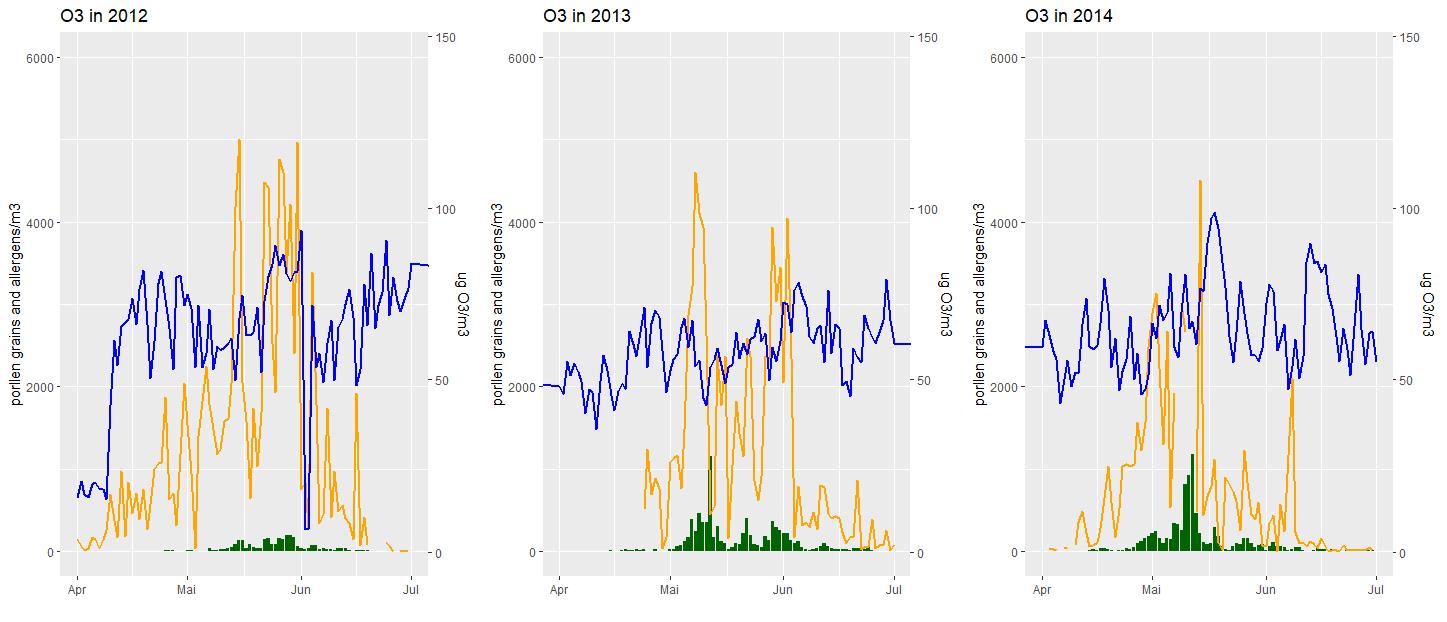
*

*
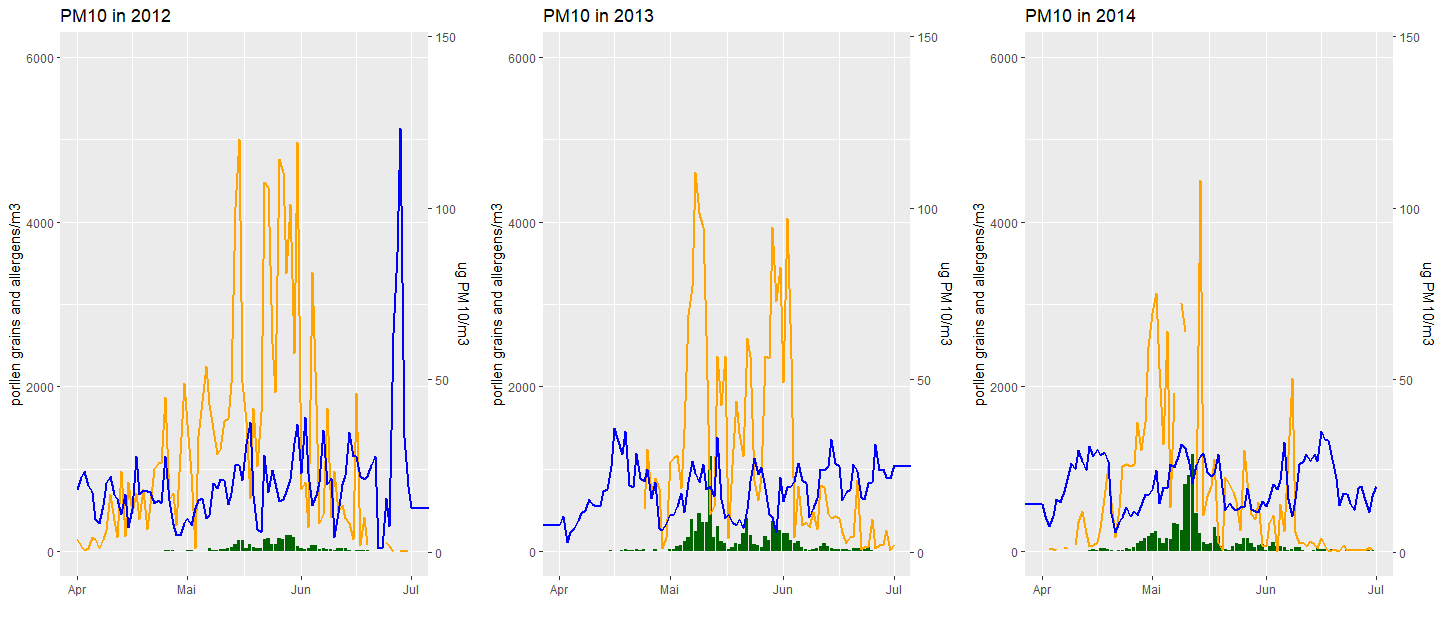
*

*
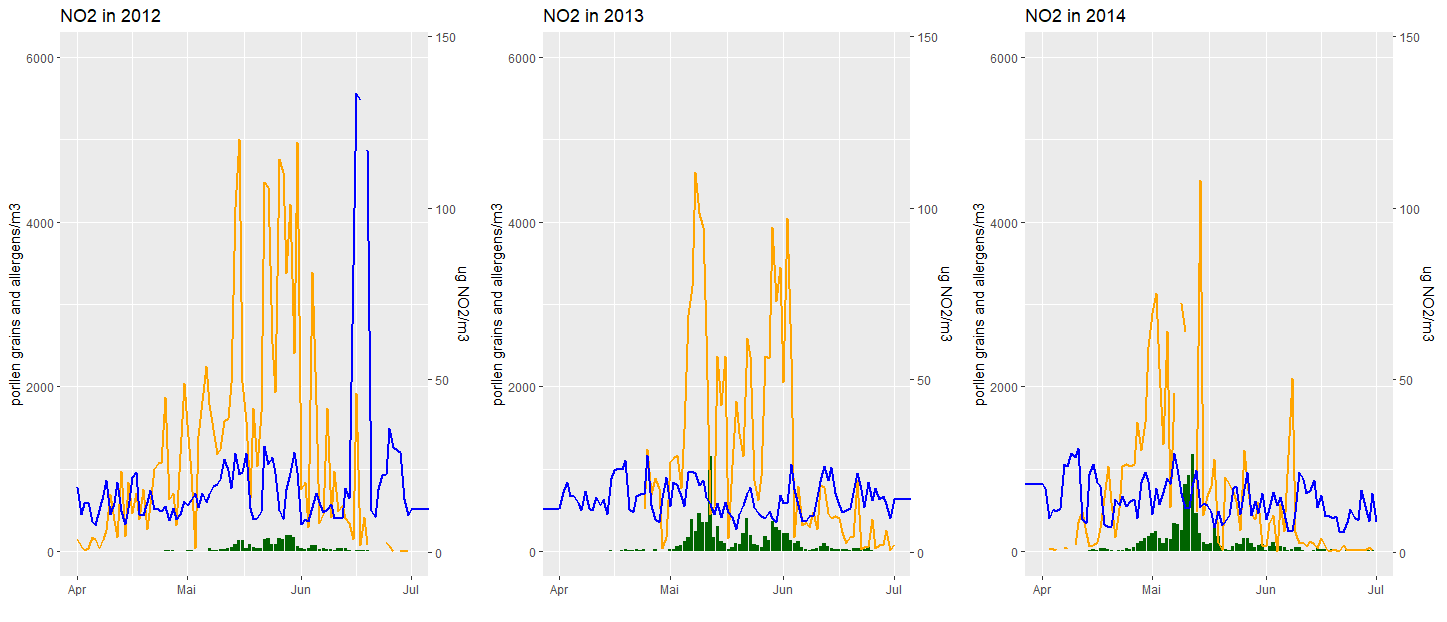
*

*
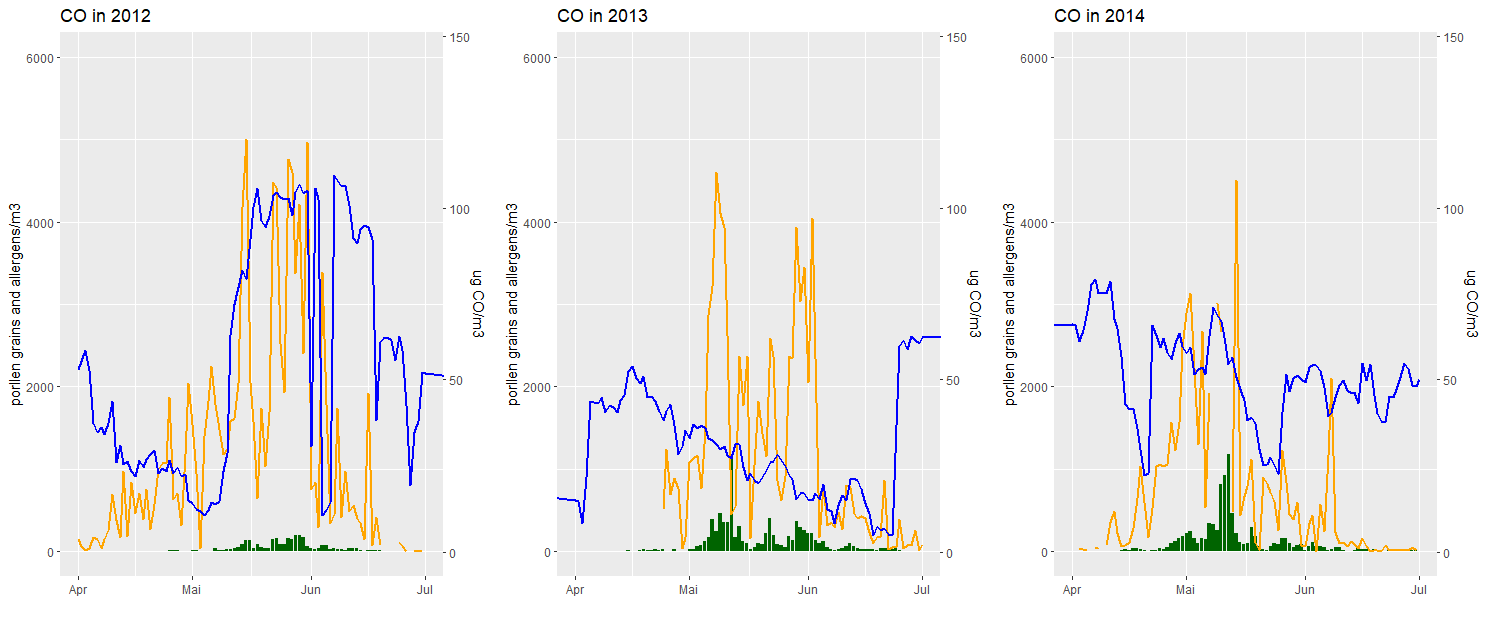
*

*Figure B. Poaceae Pollen (grains/m^3^) in green, Phl p 5 concentration in orange and SO_2,_ O_3,_ PM_10_, NO_2_ and CO pollutants concentration (in blue) during the studied period in Córdoba*

*
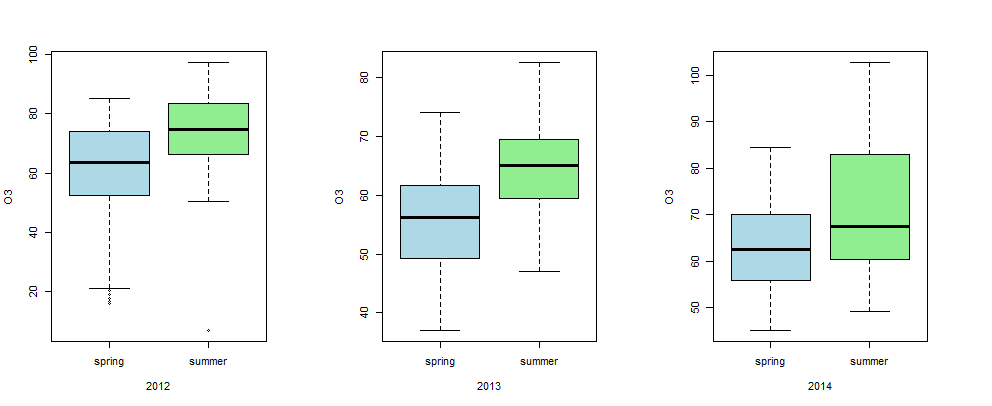

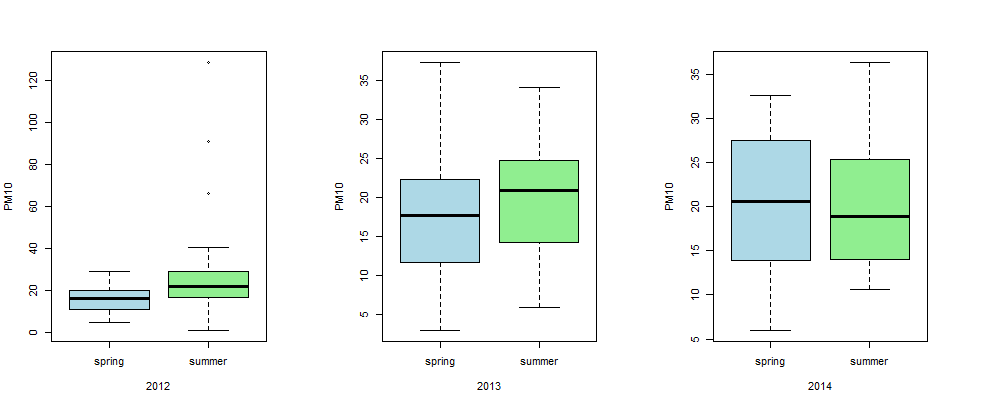

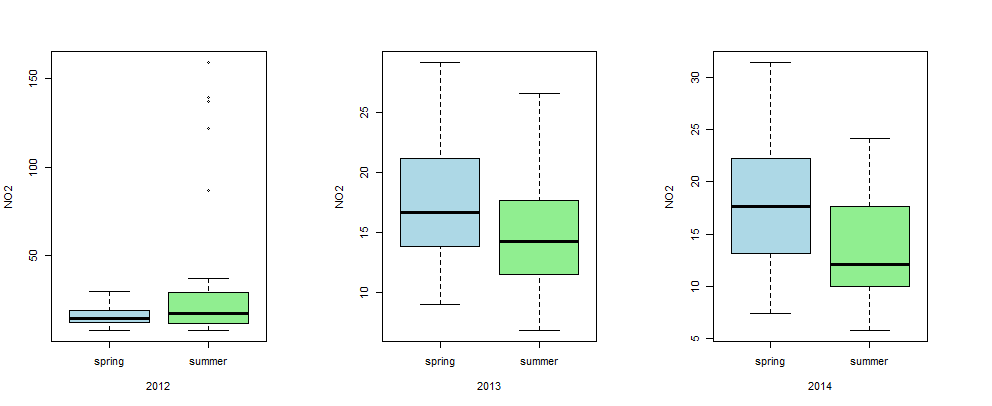

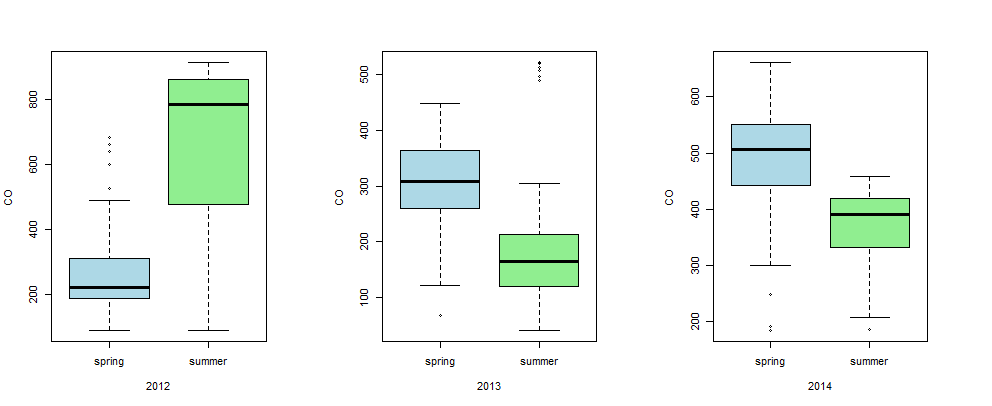

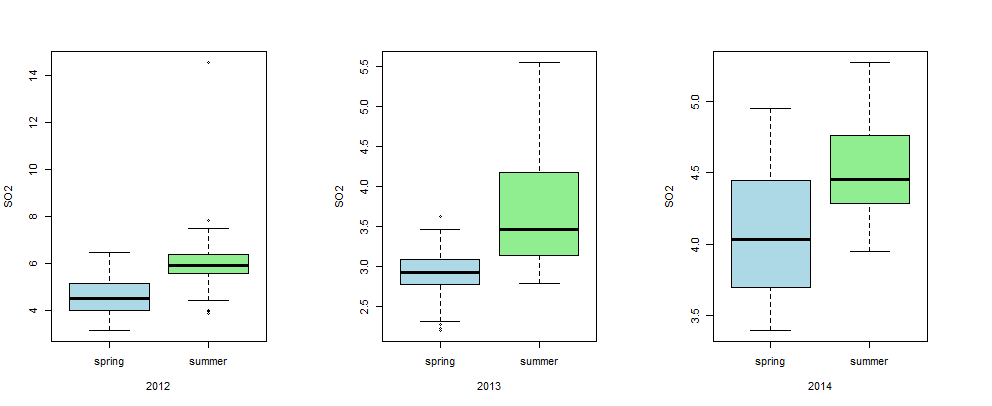
*

*Figure C. SO_2,_ O_3,_ PM10, NO2 and CO pollutants concentration according to the dates coincide with spring from 04-01 to 05-15) or early summer (from 05-16 to 07-01) during the three studied years.*

*
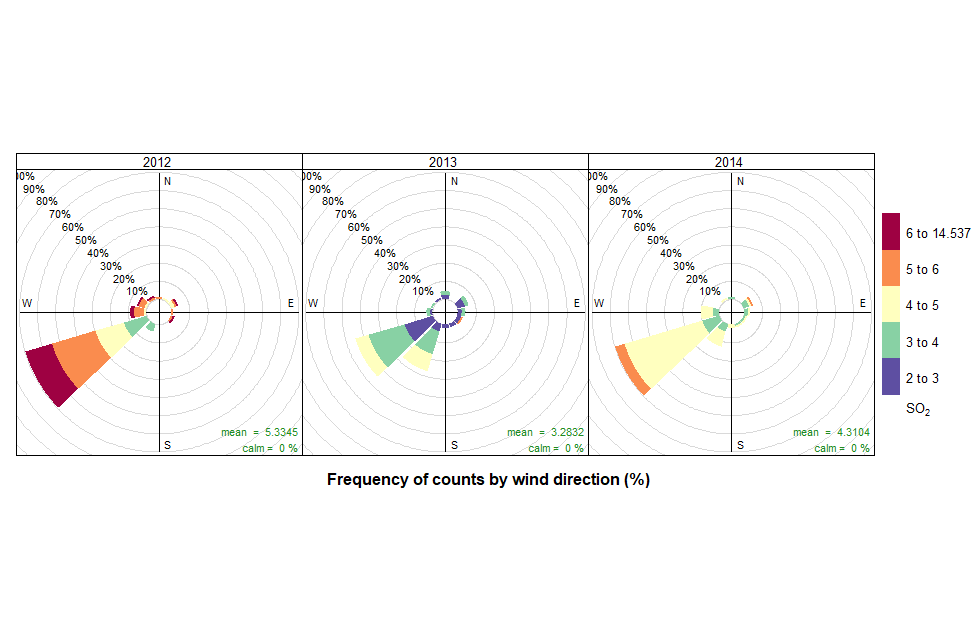

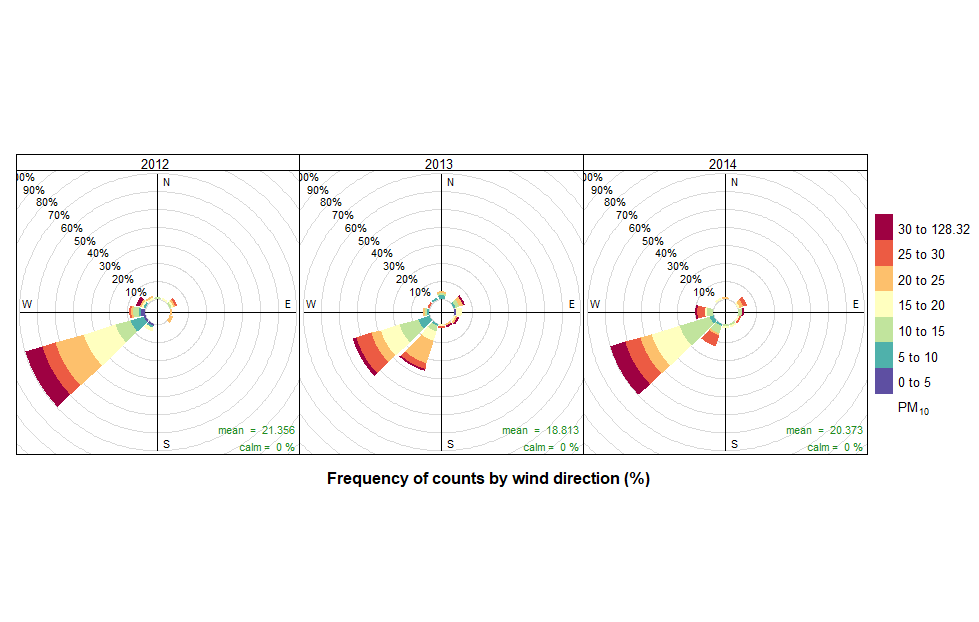

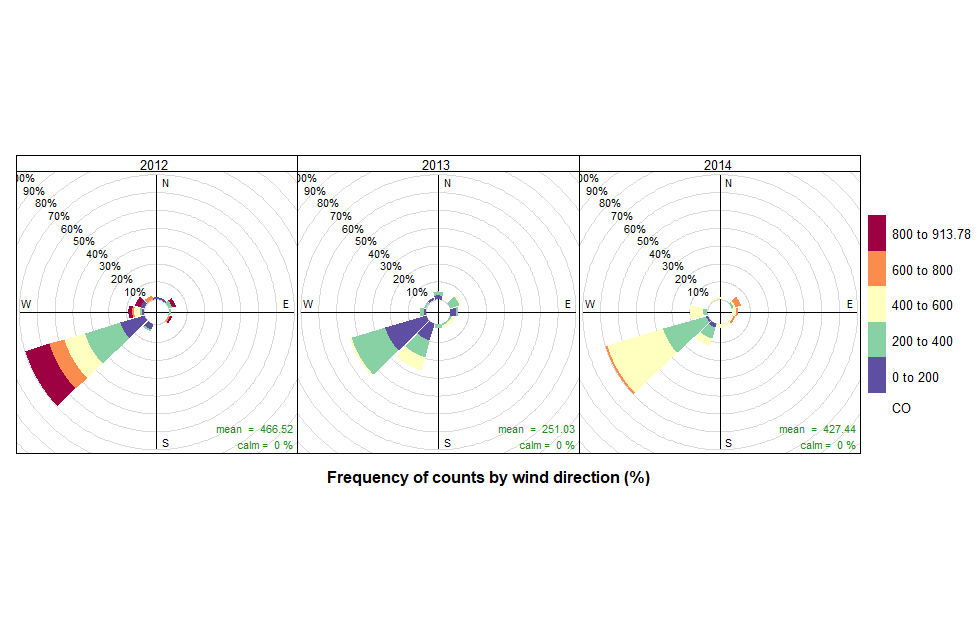
*

*Figure D. Frequency of days with the highest percentage of pollutants SO_2_, PM_10_ and CO, for each year and the prevailing wind direction.*
